# Measuring Minerals in Pseudocereals Using Inductively Coupled Plasma Optical Emission Spectrometry: What Is the Optimal Digestion Method?

**DOI:** 10.3390/foods14040565

**Published:** 2025-02-08

**Authors:** Ana C. Nascimento, Carla Motta, Andreia Rego, Inês Delgado, Susana Santiago, Ricardo Assunção, Ana Sofia Matos, Mariana Santos, Isabel Castanheira

**Affiliations:** 1 Food and Nutrition Department, National Institute of Health Doutor Ricardo Jorge, 1649-016 Lisbon, Portugal; ana.nascimento@insa.min-saude.pt (A.C.N.); carla.motta@insa.min-saude.pt (C.M.); andreia.rego@insa.min-saude.pt (A.R.); ines.delgado@insa.min-saude.pt (I.D.); susana.santiago@insa.min-saude.pt (S.S.); 2 Egas Moniz Center for Interdisciplinary Research (CiiEM), Egas Moniz School of Health & Science, Caparica, 2829-511 Almada, Portugal; rassuncao@egasmoniz.edu.pt; 3 UNIDEMI, Department of Mechanical and Industrial Engineering, NOVA School of Science and Technology, NOVA University Lisbon, 2829-516 Caparica, Portugal; asvm@fct.unl.pt; 4 Comprehensive Health Research Center (CHRC), Universidade NOVA de Lisboa, 1150-082 Lisbon, Portugal; 5 MARE—Marine and Environmental Sciences Centre, ARNET—Aquatic Research Network Associate Laboratory, NOVA School of Science and Technology, NOVA University Lisbon, 2829-516 Caparica, Portugal; isabel.castanheira2@gmail.com

**Keywords:** pseudocereals, elemental content, digestion methods, microwave, dry-ashing, graphite block acid digestion

## Abstract

Pseudocereals have gained attention due to their adaptability to different climates, high nutritional value, and suitability for gluten-free and plant-based diets. However, a challenge lies in the necessary adaptations in the diet pathways, mainly due to the lack of matrix-matching metrological tools. To address this problem, we developed a classification system to support laboratory decisions without shaped Proficiency Testing (PT) or Certified/Standard References Material. This system evaluates method performance through limit of detection (LOD), maximum uncertainty, and statistical comparison. For that matter, the mineral contents (Cu, Mn, Fe, Zn, Mg, P, Ca, K, and Na) of quinoa (*Chenopodium quinoa*), amaranth (*Amaranthus caudatus*), and buckwheat (*Fagopyrum esculentum*) were determined, using three different digestion methods, including dry-ashing, microwave, and graphite block acid digestion. A decision was reached concerning the optimal digestion method to be employed, with the results classified into three categories: (i) “rejected if results failed in two categories; (ii) “use with caution” if results were not satisfactory in one category; or (iii) “accepted”, if the results passed in all the categories. The system efficacy was exemplified by the effectiveness of dry-ashing and graphite block acid digestion by comparison with microwave digestion. Neither dry-ashing nor graphite block acid digestion can be recommended as an alternative method to the microwave digestion method when all the prioritized nutrient minerals are understudied. Although the microwave method is preferable for multi-elemental analysis, it is possible to obtain, with caution, comparable results from all the digestion methods if a higher relative combined uncertainty is defined (target uncertainty < 11%) under the assumption that this is suitable for the study.

## 1. Introduction

Nowadays, it is known that healthy eating habits are crucial to guarantee adequate nutrition patterns to maintain a good health status [1]. Appropriate daily intake of minerals is essential in preventing the risk of non-communicable diseases, such as cardiovascular diseases, certain types of cancer, or diabetes [2]. Calcium, Copper, Magnesium, Iron, Manganese, Phosphorus, Potassium, and Sodium (Cu, Mn, Fe, Zn, Mg, P, Ca, K, and Na) are included in the group of prioritized nutrients to cover, as they are essential for maintaining several vital functions [3]. In the European Prospective Investigation into Cancer and Nutrition (EPIC) study, these minerals have been prioritized as key nutrients to monitor and assess nutrient intake’s impact on health status [4].

Pseudocereals have been gaining particular interest as sources of minerals for gluten-free diets [5,6,7]. Pseudocereals such as quinoa (*Chenopodium quinoa*) and amaranth (*Amaranthus caudatus*) are part of the diet in the Andean region of Bolivia, North of Argentina, and Peru [8,9]. Moreover, they are included as part of vegetarian diets and healthier consumer patterns across other regions, such as Japan, India, Australia, and Europe [5,6,8,10]. Additionally, the National Aeronautics and Space Administration (NASA) has classified quinoa as an emerging crop with excellent nutritional properties for long-term human space missions [11]. Common buckwheat (*Fagopyrum esculentum*) is another pseudocereal comparable to quinoa and amaranth [12]. Its use is due to its high protein content, which exceeds wheat, maize, and rice [13,14].

The successful use of pseudocereals in gluten-free diets has been demonstrated in several studies and was recently reviewed by several authors [5,6,7,8]. These studies have shown that a well-balanced intake of Ca and Fe can be achieved when these pseudocereals are included in the diet, replacing other gluten-free ingredients. Several publications have highlighted their importance in celiac patients’ diets due to their rich nutrient profile, making them a significant source of essential minerals. So, the World Gastroenterology Organisation has recognized these pseudocereals as suitable for gluten-free diets [15]. Their rheological properties, sensory characteristics, nutrient profile, and stability allow gluten-free formulations based on quinoa or amaranth to achieve a texture similar to corn-based formulations. In parallel, their taste, smell, and flavor enhance consumer preferences [16]. Therefore, accurate determination of the elemental content in pseudocereals is critical for evaluating nutritional benefits and confirming that their consumption supports an adequate nutrient intake [17].

The analytical method used for food mineral analysis, Inductively Coupled Plasma Optical Emission Spectrometry (ICP-OES), stands out as a multi-element analysis technique with several advantages over other techniques.

From the perspective of laboratory analysis, the most notable advantage of ICP-OES is that it allows for the determination of a considerable number of elements in a large number of samples in a short time, with no compromise of precision or detection limits and can provide analytical results in a short time [3]. This is in contrast to the flame and graphite furnace atomic absorption techniques, where the lamp is specific to a limited number of elements; thus, only one (or a few) element can be measured at a time. In conjunction with a short analysis time and simple sample preparation, ICP-OES offers the opportunity to obtain a very high sample throughput in the laboratory [18].

Several digestion methods are available for sample preparation, such as dry-ashing, wet-ashing, and microwave digestion [19,20,21,22,23,24,25,26]. Yet, some digestion methods that precede instrumental analysis are time-consuming; for instance, dry-ashing is slower than microwave digestion, which takes hours to days longer [19,20,21,22]. Additionally, cross-contamination can occur using dry-ashing, as is an open method, and some elements can be present in the laboratory environment or in reagents [27]. Nevertheless, more straightforward analytical methods like dry-ashing may be useful in laboratories lacking the more complex and expensive equipment for other digestion methods.

In previous studies, our team employed microwave digestion for mineral analysis in different pseudocereals [28]. Dry-ashing and graphite block acid digestion are methods commonly used in many laboratories instead of microwave digestion. However, they need validation due to the complexity of pseudocereals matrices and the lack of Certified Reference Materials (CRM) and Proficiency Testing (PT) Schemes. In this context, there are several approaches available for evaluating laboratory performance. Our group has previously applied the Six Sigma approach to different sources of raw data to investigate the possibility of using these methods to aggregate data from different sources, demonstrating the complementarity between this approach and the use of a data quality evaluation system applied to nutrient data [29].

Method performance is crucial in selecting analytical approaches for determining components in food matrices [30]. The European Union (EU) has introduced new legislation emphasizing method performance and target uncertainty in analytical procedures. This ensures methods are reliable, accurate, and validated, supporting harmonized practices, compliance with regulations, and credible scientific data [31,32]. This allows laboratories to decide on a fit-for-purpose rather than adhering strictly to a standardized method.

Therefore, the purpose of this study was to evaluate the performance of three digestion methods, including dry-ashing, microwave, and graphite block acid digestion, for the analysis of mineral contents (Cu, Mn, Fe, Zn, Mg, P, Ca, K, Na) in pseudocereals, namely in quinoa (*Chenopodium quinoa*), amaranth (*Amaranthus caudatus*) and buckwheat (*Fagopyrum esculentum*). The performance of the different digestion methods was assessed using an approach based on the limit of detection, uncertainty, and statistical comparability test. For that matter, a classification system to support the decision on the best digestion method to apply was developed, categorizing the results into three main categories: (i) “rejected”, if results failed in two categories; (ii) “use with caution”, if results were not satisfactory in one category; or (iii) “accepted”, if the results passed in all the categories.

## 2. Materials and Methods

The mineral content (Cu, Mn, Fe, Zn, Mg, Ca, P, Na, and K) in samples of three pseudocereals, namely quinoa, amaranth, and buckwheat, was determined by ICP-OES. Before analysis, the samples were digested using three distinct methods: dry-ashing, microwave digestion, and graphite block acid digestion.

### 2.1. Sampling and Sample Preparation

Samples of quinoa and amaranth were obtained from the Cooperative of Producers (CAUQUEVA- Tilcara, Jujuy, Argentina). Buckwheat was purchased from a supermarket in Portugal. Primary samples were collected according to a selective sample plan. Five samples of each material were collected: for amaranth only once and for quinoa and buckwheat over two consecutive years.

All samples collected were ground and homogenized using a high-speed grinder (Retsch™ Knife Mill GRINDOMIX GM 200, Haan, Germany) equipped with titanium knives to prevent contamination. Individual samples were stored separately in aluminum foil vacuum bags for proper preservation at room temperature until analysis.

### 2.2. Reagents and Chemical Standards

All reagents were of a high analytical grade. Deionized water of level I (Millipore, Burlington, MA, USA), as defined in ISO 3696:1987 [33] and hydrogen peroxide (30%; Merck, Darmstadt, Germany) solutions of ultrapure grade were used. Also, concentrated nitric acid (65%; Merck, Darmstadt, Germany), first distilled in an acid distillation system (model subPUR; Milestone, Shelton, CT, USA), was used.

Working multi-element standard solutions were prepared from mono-element high purity ICP stock standards (Cu, Mn, Fe, Zn, Mg, Ca, P, Na, and K) containing 1000 mg/L of each element (Merck, Darmstadt, Germany).

Working standard solutions, sample dilutions, and blanks were prepared with a 2% concentration solution of nitric acid. A nitric acid solution (2–4%) concentration was used to wash up the ICP-OES sample introduction system.

### 2.3. Sample Digestion Procedures

Three digestion procedures, dry-ashing (DA), microwave (MW), and graphite block acid digestion (DP), were applied as described below.

#### 2.3.1. Dry-Ashing

Foodstuffs samples (0.5 g) were placed in high-form crucible platinum and burned to ashes using a Bunsen burner. Ash samples were placed in a muffle furnace (Heraeus, M110, Heraeus Instruments, Hanau, Germany), at 470 °C for 40 min. The white ash residues obtained were dissolved with water (1 mL) and nitric acid 65% (0.5 mL) and heated in a water bath for 3 min without completely dry. These solutions were transferred to a volumetric flask (25 mL; Class A glassware) and completed with deionized water. Finally, samples were filtered with a paper filter (Whatman, N°41, Ashless, Whatman, Maidstone, UK), and transferred to analyzing tubes. The methods of the Association of Official Agricultural Chemists (AOAC), AOAC Official Method 984.27 and AOAC Official Method 985.35 were used as guidance in the development of this method [34,35].

#### 2.3.2. Microwave Digestion

Samples were weighted (0.5 g) to proper Teflon digestion vessels. A mixture of concentrated nitric acid (4 mL), hydrogen peroxide (1 mL) and deionized water (3 mL) was carefully added. Vessels were closed correctly and introduced into the microwave oven. Digestions were undertaken using a closed-vessel microwave digestion system (Milestone ETHOS 1 Series). A previously optimized five-stage microwave digestion program [28], with a maximum temperature of 210 °C and a minimum of 90 °C, with times varying from 6 to 12 min, was chosen. After digestion, vessels were cooled to room temperature and opened. The digested samples were diluted up to 25 mL with deionized water and transferred to analyzing tubes.

#### 2.3.3. Graphite Block Acid Digestion

Samples were weighted (0.5 g) in polypropylene tubes and kept overnight in nitric acid (4 mL). In the day after, hydrogen peroxide (1 mL) was added. The digestion step was carried out in a graphite heating block system at high temperature (SCP Science, DigiPREP MS, Baie-d’Urfé, QC, Canada), using a stepwise approach with a temperature and time optimized program (15 min at 35 °C; 25 min at 45 °C; 80 min at 80 °C). Digestion samples were then cooled, diluted up with deionized water to 25 mL and transferred to analyzing tubes.

### 2.4. ICP-OES Analysis

An inductively coupled plasma optical emission spectrometer with radial and axial configuration (ICP-OES Thermo iCAP 6000 series, Thermo Scientific, Waltham, MA, USA) was used for the analysis of Cu, Mn, Fe, Zn, Mg, Ca, P, Na and K in DA, MW and DP digested samples. ICP-OES instrumental setting conditions are presented in Table 1. The ISO 11885:2007 was used as guidance for developing this method [36].

### 2.5. Quality Assurance Procedures

Following the European Food Information Resource (Eurofir) guidelines, an internal quality control program was implemented [37]. These criteria encompass the use of titanium knives to avoid sample contamination (sample handling) and of validated analytical methods, including digestion of samples, for the preparation and analysis of the samples (methods of analysis).

Two independent quality control samples were included in the runs at each group of 10 samples with an acceptance criterion of ±10% for both. The repeatability of the measurement procedure was evaluated by triplicate analysis.

To assess possible contaminations, blank solutions were prepared containing the same reagents and using the same procedure as the samples and standards.

Quality parameters were determined for all the minerals and digestion methods in the pseudocereal samples under study.

During method validation, the linearity of the calibration curves was assessed, and the working range of the calibration curve was determined.

Sensitivity, limits of detection (LODs), and limits of quantification (LOQs) were determined using Thompson and EFSA approaches [38,39]. LODs and LOQs utilized three times and ten times the standard deviation of the blank analytical signal divided by the slope, respectively [38]. Precision values were expressed as coefficient of variation (CV).

To evaluate the accuracy and precision of the digestion methods, a Standard Reference Material (SRM), NIST SRM 1548a Typical Diet (National Institutes of Standards and Technology, Gaithersberg, MD, USA) was used and prepared in the same way as the samples.

Accuracy was determined by comparing the measured concentration with the certified value and was expressed as percentage recovery (Rec. %). Satisfactory precision and accuracy were required to be within <20% and 80–120% for all elements, respectively [40].

Intra and interlaboratory performance for digestion methods was monitored through the analysis of the SRM. Additionally, satisfactory participation in regular PT schemes launched by accredited PT providers showed laboratory performance.

### 2.6. Calculation of the Uncertainty as a Function of Concentration

Thompson and co-authors introduced the concept of uncertainty function (*u_f_*) (maximum standard uncertainty), which tells us how measurement uncertainty changes with the concentration of an analyte [38,39]. For the calculation of uncertainty as a function of concentration, Equation (1) was used.

(1) $$u_{f}=\sqrt{{\left(LOD/2\right)}^{2}+{\left(aC\right)}^{2}}$$

where *u_f_*—is uncertainty *u* as a function (*f*) of the concentration *C* of the analyte; *LOD*—is limit of detection of the method; *C*—is mass fraction of analyte; and a—is the numeric factor to be used for the range of concentrations, as described in the European Legislation and Codex [41].

According to Thompson and Wood [38], this concept was considered sufficiently robust and suitable for evaluating the performance of analytical methods. As such, it was decided that it was to be used as a quality criterion.

### 2.7. Classification System for the Digestion Methods

Since the bias associated with each digestion method could not be determined due to the lack of matrix-matching CRM/SRM and PT schemes, additional statistical tests were applied. A procedure was used based on three parameters, namely, LOD, maximum uncertainty, and statistical comparison. In this exercise, the microwave digestion method was considered the reference method. This approach was based on FDA guidance and Bhagwat et al.’s [42] approach, considering the final use of data.

The LOD is relevant for evaluating the method’s suitability and applicability/comparability in measuring pseudocereal elemental content [22]. Given the limited number of validated methods for analyzing elemental content in pseudocereals and the potential for matrix effects in ICP-OES analysis [43], we adhered to Thompson’s concept [38] in our work. Also, we considered the LOD to be one of the critical parameters for defining the method’s comparability.

Therefore, LOD is the exclusion criteria, and results can be categorized into three main categories: (i) “rejected”, if results are below LOD or failed in two categories; (ii) “use with caution”, if results were not satisfactory in one category; or (iii) “accepted”, if the results passed in all the categories.

Subsequently, if the results are above LOD, uncertainty is estimated by Equation (1) and compared with a target uncertainty (11%). After statistical inference is applied.

The digestion method can be used if both categories (uncertainty and statistical inference) are within their acceptance criteria. If one fails, the digestion method can be applied with caution; otherwise, it should be rejected.

### 2.8. Statistical Analysis

All samples were analyzed in triplicate, and the results were expressed as a mean and standard deviation (SD). The mean values of elemental content for the three digestion methods were compared by analysis of variance (ANOVA). When the overall result was statistically significant, the Tukey–Kramer multiple comparison test was conducted to identify which digestion methods were significantly different. Homogeneity of variances was tested using Cochran’s and Levene’s tests, and the results were undertaken using the Kruskal–Wallis non-parametric test when variances were heterogeneous. All statistical tests were conducted using Statistica v. 8 software (Statsoft Ibérica, Lisboa, Portugal). Differences were considered statistically significant at a *p*-value below 0.05.

## 3. Results and Discussion

### 3.1. Accuracy and Precision Assessment of Digestion Methods

The verification of LOD and LOQ estimated for the nine elements in pseudocereals, obtained from the three different digestion methods (DA, MW, and DP), was performed by checking performance characteristics (accuracy and precision) for elements present in NIST 1548a reference material (Table 2).

The detection limit and quantification limit values of the analyzed elements were found to be higher when DA decomposition was used. Major differences were observed for Cu, Fe, Ca, Na, and P. This may be explained by the possible contaminations in the DA decomposition process, as described by [24].

The CV was less than 20% for all minerals and the percentage recovery (Rec. %) was between 80 and 120% for all elements. The agreement between experimental and certified results for NIST 1548a indicates that the analytical procedures applied suit all digestion procedures.

### 3.2. Uncertainty Evaluation

Table 3 presents values for experimental uncertainty, expressed as combined uncertainty (*u_c_*). These values were obtained from the exploitation of single validation as described by Coelho et al. [44].

Uncertainty ranged between 0.08 and 0.11. The lowest value was observed for Ca and Zn determination when DP was used. The highest experimental uncertainties were achieved for K using DP, and Cu, using DA, which were possibly caused by eventual cross-contaminations. Both methods were carried out in an open vessel, and environmental laboratory cross-contamination could have occurred from air dust or metal contaminations in the Cu case due to the use of various metal materials in the DA method, which is avoided using the microwave, a closed method [45].

Table 4 presents the values of the uncertainty *u_f_* as a function of the concentration C of the analyte calculated according to Equation (1).

The combined standard uncertainty values (*u_c_*) were compared with the target uncertainty values (*u_f_*) calculated by Equation (1). The uncertainty value was similar in the three digestion methods, but it was higher in magnesium. Phosphorus and potassium for all samples wer DA, MW and DP analyzed when DP was used. These values were used to evaluate the method’s applicability particularly in measuring lower concentrations.

For all three methods, the combined standard uncertainty (*u_c_*) in iron, zinc, magnesium, calcium, phosphorus, and potassium is less than the maximum allowable standard uncertainty (*u_f_*). We can infer that the three methods are equivalent in terms of uncertainty to assist in the determination of minerals by ICP-OES in quinoa, amaranth, and buckwheat. Concerning uncertainties and reproducibility for each method, all three methods applied for mineral content are in good accordance with the acceptance criteria [46].

### 3.3. Determination of Elemental Contents in Pseudocereals: Comparison of Digestion Methods

Table 5 presents elemental contents (mg/100 g) of pseudocereals obtained by DA, MW, and DP methods, considering microwave as the reference method for statistical inference.

In Cu analysis, there were no significant differences between the digestion methods studied for amaranth and quinoa. However, significant differences were observed for buckwheat when the MW method was used to digest the samples (DA with *p* = 0.006; DP with *p* = 0.002).

Mn and Fe analysis showed no significant differences among the three different digestion methods studied for amaranth and buckwheat. Quinoa presented significant differences between DP and MW (*p* = 0.006 for Mn and *p* = 0.014 for Fe).

Zn amounts were not significantly different in all the digestion methods studied for quinoa and buckwheat. Amaranth DA was significantly different from amaranth MW (*p* = 0.022).

Mg and P values were not significantly different in all the digestion methods studied for all the samples analyzed, presenting for all comparisons a *p*-value upper than 5%.

For Ca, the only significant differences were found in buckwheat when DP was applied (*p* = 0.042). For the other matrix, no significant differences were found (*p* > 0.05).

In K, the values were statistically similar in all digestion methods studied for quinoa and buckwheat. However, in amaranth, DP showed significant differences with MW (*p* = 0.048).

Our results suggest that the equivalence of the method to assist in the analysis depends on the component and the digestion method itself. These findings agree with Althundag et al. [22] for Cu, Mn, Zn, and Fe in dry foods, where the comparison of dry, wet, and microwave digestion methods showed no significant differences in the results obtained for the three methods mentioned.

### 3.4. Assessment of Digestion Methods Based on the Criteria Developed to Evaluate the Comparability with Microwave Digestion Method

A procedure was used based on three parameters namely LOD, maximum uncertainty and statistical comparison was developed since no metrological tools—CRM/SRM or PT Schemes—were available for pseudocereals. The aim was to evaluate if other digestion methods would provide the same result as the microwave digestion method.

The statistical inference concept was applied to the analytical data under repeatability conditions, being an acceptance criterion defined for each category.

The strategy understudy was developed considering the classical method validation and comparability approach, as described in the food composition database quality evaluation system [47]. This approach was adopted due to the common final purpose of the data.

The results of this evaluation are presented in Table 6.

**Figure 1:**
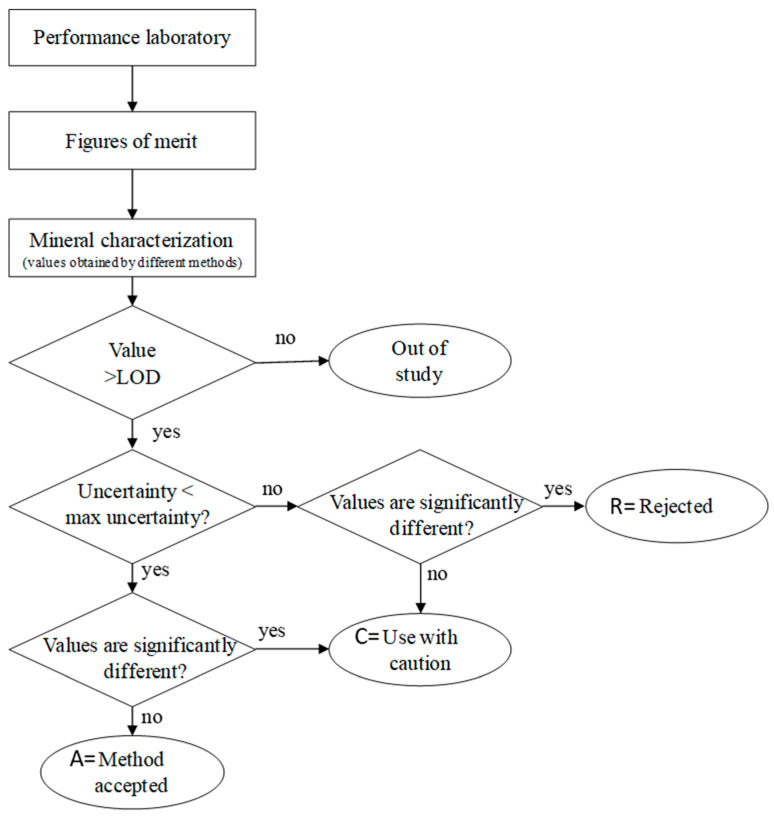
Diagram describing the process and classification system developed and applied in this study.

An overview of the classification system describing how parameters and acceptance criteria were combined to evaluate the method’s comparability with MW is present in Figure 1.

Considering LOD as an essential parameter in ICP analysis, all the methods are suitable for measuring the elemental content in pseudocereals. The three methods have an appropriate performance for Mg and P, with good precision and accuracy for all pseudocereals in the study, suggesting that analytical procedures applying DA, DP, or MW are reliable. However, some pitfalls were observed for Cu where higher uncertainties were estimated for all pseudocereals. Critical differences for Cu values in foods were also reported in the literature when DA is used [26]. Furthermore, Cu in buckwheat analyzed by DA exhibited uncertainty slightly above the limit and significant differences with MW.

Concerning K, significant differences were observed using DP in amaranth. According to these results, DP should be used with caution when determining K in amaranth.

Fe and Mn present the same pitfall for DP in quinoa; as such, DP should be used with caution for the determination of Fe and Mn in quinoa, while DA can be used. No significant differences were observed between DA and DP for amaranth and buckwheat. Similarly, no differences were observed between dry and wet ashing for cereals (rice, maize, and wheat) by Akinyele et al. [26].

Zn presents significantly lower values when the digestion method is DA in amaranth, while Ca presents lower values in DP for buckwheat. The most appropriate method for determining both elements should be MW. As expected, the sodium content is negligible in raw pseudocereals (<LOD).

In selecting digestion methods, the laboratory has to consider which minerals and pseudocereals are under analysis and choose a method that provides acceptable results. Our study concludes that DA is more time-consuming, whereas MW is a simpler and faster method. Also, the closed-vessel microwave digestion system avoids losses due to volatilization more effectively and prevents sample contamination problems, which agrees with several authors [20,23]. On the other hand, dry-ashing is referred to in the literature as a method with small risks involved and equipment simplicity. Furthermore, this method permits the utilization of a higher quantity of samples, thus increasing the concentration of the analyte. This is crucial when quantifying a mineral in trace quantities [26]. In addition, MW requires costly instrumentation, which needs proper maintenance and is not available in all laboratories in which pseudocereals are studied. The selection of the analytical protocol depends on different applications and purposes.

According to our results, using DA or DP as alternative digestion methods of MW is adequate for almost all minerals. If higher uncertainties could be accepted for the purpose (e.g., instrumental methods used with different LODs), DA could be considered as an acceptable method for quinoa and amaranth, and with caution for buckwheat regarding Cu. In contrast, DP could be acceptable for all the pseudocereals under study.

The results of this study are consistent with those of Poitevin, E. et al., who carried out a comparative analysis of conventional digestion methods and microwave-assisted digestion techniques prior to ICP-OES analysis. The study concluded that the MW method is particularly suitable for multi-elemental analysis, as it proved to be an accurate technique for determining nutritional minerals, which is a critical aspect in the context of food labeling [48].

Considering the health benefits of pseudocereals and their suitability for gluten-free and plant-based diets, it is essential to use suitable analytical methodologies for their mineral assessment, especially given their increasing popularity and the growing recommendations for more sustainable diets. As pseudocereals are valued for their high mineral content, and translating elemental content into intake can involve several sources of error, including those from analytical methods, the digestion methods will be considered equivalent if the final results do not impact the estimation of intake and its associated tolerance interval [30]. Additionally, selecting a digestion method should reflect the Eurachem statement: “the judgment of method suitability for its intended use is equally important to the evaluation process” [47].

## 4. Conclusions

Pseudocereals align with current guidelines for sustainable and plant-based diets, which recommend prioritizing diverse whole foods and emphasize minimizing the intake of processed foods and animal products while promoting environmentally friendly practices like reducing food waste and opting for locally sourced and seasonal ingredients. Therefore, several aspects of analytical quality and quality control are essential when the elemental contents of pseudocereals are used for health status monitoring over time and for multicenter epidemiological studies.

Multi-element techniques for the quantification of inorganic food components are very useful under the assumption that organic matter is completely destroyed before instrumental analysis. The developed approach based on three criteria (LOD, uncertainty, and statistical comparison) was successfully applied, allowing a suitable selection of the digestion methods to give acceptable results.

This is the first study that has compared three different digestion methods for pseudocereals before mineral analysis using ICP-OES. The choice of digestion method should consider the requirements for precision and accuracy.

Neither DA nor DP can be recommended as an alternative method to MW when all the prioritized nutrient minerals are understudied. Although the MW method is preferable for multi-elemental analysis, it is possible to obtain, with caution, comparable results from all the digestion methods if a higher uncertainty is defined (target uncertainty <0.11 (11%)) under the assumption that this is suitable for the study.

The findings of this study will benefit those engaged in the scrutiny of data, particularly those involved in the compilation of food composition databases.

A suitable methodology has been developed to assess the quality of the analytical data entered into the Food Composition Database. The described procedure complements the data quality system that is already in place. The strategy applied fills the gap between several data quality systems since it focuses on component extraction.

The methodology was applied to various pseudocereals, enabling the determination of eight priority minerals identified by the Food Composition Communities. The study emphasized the relevance of the method for nutritional analysis and public health applications.

## Figures and Tables

**Table 1:** ICP OES: Instrumental operating conditions.

ICP OES—Thermo ICAP 6000 Series	Operating Conditions
Auxiliary gas flow (L/min)	0.5
Radio frequency power (W)	1200
Nebulization pressure (psi)	on
Peristaltic pump speed (rpm)	50
Pump stabilization time (s)	5
Integration time in the UV and Visible (s)	15 and 10
Plasma view mode	Axial and Radial ^a^

^a^ Axial—Cu, Mn; Radial—Mg, Ca, Na, P, K.

**Table 2:** Laboratory performance: accuracy and precision values results found in the Standard Reference Material 1548a Typical Diet using different digestions methods.

Parameter	Work Range (mg/kg)	Λ (nm)	LOD (mg/kg)			LOQ (mg/kg)				Accuracy and Precision		
			DA	MW	DP	DA	MW	DP	Certified Value ± U ^(i)^	Certified Found (mg/100g) ^(ii)^ (CV and Recovery, %)		
										DA	MW	DP
Cu	0.02–0.2	324.754	0.022	0.0058	0.00076	0.039	0.018	0.0023	0.23 ± 0.02	0.23 ± 0.02 (9; 98)	0.23 ± 0.01 (6; 100)	0.23 ± 0.02 (9; 98)
Mn		259.373	0.0065	0.0028	0.00045	0.020	0.0084	0.0014	0.57 ± 0.02	0.56 ± 0.01 (3; 98)	0.56 ± 0.01 (3; 98)	0.56 ± 0.01 (3; 98)
Fe	0.05–0.5	259.940	0.017	0.0065	0.018	0.052	0.020	0.053	3.53 ± 0.38	3.25 ±0.21 (7; 93)	3.40 ± 0.14 (4; 97)	3.25 ± 0.21 (7; 93)
Zn		213.856	0.010	0.0062	0.018	0.030	0.019	0.054	2.46 ± 0.18	2.49 ± 0.26 (11; 99)	2.45 ± 0.21 (9; 98)	2.49 ± 0.26 (11; 99)
Mg	1–10	279.553	0.12	0.12	0.015	0.36	0.35	0.045	58.0 ± 2.7	57.0 ± 1.4 (2; 98)	59.5 ± 2.1 (4; 103)	57.0 ± 1.4 (2; 98)
Ca	2–20	184.006	0.55	0.13	0.16	1.0	0.39	0.47	197 ± 11	197.0 ± 1.4 (1; 100)	194.5 ± 2.1 (1; 99)	197.0 ± 1.4 (1; 100)
P		178.284	0.14	0.055	0.065	0.41	0.17	0.20	349 ± 24	334.5 ± 6.4 (2; 96)	344.0 ± 4.2 (1; 99)	334.5 ± 6.4 (2; 96)
Na		589.592	0.55	0.16	0.16	0.95	0.48	0.47	813 ± 94	826.0 ± 19.8 (2; 102)	800.0 ± 33.9 (4; 98)	826.0 ± 19.8 (2; 102)
K	2.5–25	769.896	0.52	0.25	0.37	1.1	0.74	1.1	697 ± 13	687.5 ± 4.9 (1; 99)	690.0 ± 8.5 (1; 99)	687.5 ± 4.9 (1; 99)

DA—Dry-ashing; MW—microwave; DP—graphite block acid digestion; R > 0.9995 for all minerals analyzed. ^(i)^ Nist SRM 1548a Typical Diet. National Institutes of Standards and Technology. Gaithersberg. MD. USA; ^(ii)^ SD from the average of triplicate analysis.

**Table 3:** The combined standard uncertainty (*u_c_*) values of the methods based on the exploitation of the in-house validation data.

Digestion Method	Minerals							
	Cu	Mn	Fe	Zn	Mg	Ca	P	K
DA	0.11	0.088	0.089	0.095	0.097	0.089	0.087	0.098
MW	0.088	0.092	0.082	0.078	0.091	0.97	0.086	0.099
DP	0.096	0.088	0.092	0.082	0.091	0.076	0.092	0.10

DA—dry-ashing; MW—microwave; DP—graphite block acid digestion.

**Table 4:** Maximum standard uncertainty (*u_f_*) calculated according to Equation (1).

Minerals	Digestion Method	*u_f_* (mg/100 g)		
		Quinoa	Amaranth	Buckwheat
Cu	MW	0.053	0.046	0.032
	DA	0.059	0.047	0.036
	DP	0.046	0.047	0.037
Mn	MW	0.17	0.14	0.10
	DA	0.16	0.13	0.11
	DP	0.15	0.14	0.10
Fe	MW	0.48	0.88	0.25
	DA	0.45	0.82	0.26
	DP	0.42	0.90	0.25
Zn	MW	0.26	0.51	0.17
	DA	0.29	0.42	0.21
	DP	0.28	0.46	0.16
Mg	MW	17	21	20
	DA	16	21	19
	DP	18	26	21
Ca	MW	3.9	15	1.9
	DA	4.0	15	1.7
	DP	3.6	17	1.5
P	MW	41	48	33
	DA	38	47	33
	DP	43	60	37
K	MW	59	48	41
	DA	57	49	40
	DP	60	59	44

DA—dry-ashing; MW—microwave; DP—graphite block acid digestion.

**Table 5:** Elemental composition of pseudocereals using different digestion methods (mg/100 g).

		Minerals							
Pseudocereal	Digestion Method	Cu	Mn	Fe	Zn	Mg	P	Ca	K
Quinoa	DA	0.59 ^a^ ± 0.03	1.60 ^ab^ ± 0.01	4.47 ^ab^ ± 0.08	2.95 ± 0.26	161.3 ± 1.0	377.5 ± 2.9	40.4 ^a^ ± 0.6	569.8 ± 12.8
	MW	0.51 ^ab^ ± 0.02	1.73 ^a^ ± 0.11	4.83 ^a^ ± 0.01	2.588 ± 0.002	173.9 ± 9.6	413.8 ± 16.4	38.5 ^ab^ ± 2.5	586.7 ± 20.8
	DP	0.46 ^b^ ± 0.02	1.49 ^b^ ± 0.05	4.22 ^b^ ± 0.16	2.83 ± 0.04	176.0 ± 3.1	431.2 ± 12.6	36.2 ^b^ ± 0.7	603.5 ± 18.5
Amaranth	DA	0.47 ± 0.04	1.35 ± 0.03	8.20 ± 0.31	4.15 ^a^ ± 0.15	207.5 ± 9.1	474.2 ± 20.8	153.6 ± 5.9	490.4 ^a^ ± 1.9
	MW	0.464 ± 0.001	1.38 ± 0.04	8.769 ± 0.001	5.11 ^b^ ± 0.50	210.8 ± 5.8	480.2 ± 18.2	150.5 ± 12.8	482.9 ^a^ ± 27.5
	DP	0.474 ± 0.004	1.44 ± 0.01	8.96 ± 0.15	4.61 ^ab^ ± 0.01	262.1 ± 9.7	599.5 ± 20.9	170.5 ± 3.3	589.5 ^b^ ± 21.0
Buckwheat	DA	0.36 ^a^ ± 0.01	1.073 ± 0.028	2.58 ± 0.09	2.07 ± 0.10	193.9 ± 15.1	326.4 ± 6.7	17.3 ^a^ ± 0.2	403.2 ± 9.6
	MW	0.32 ^b^ ± 0.01	0.982 ± 0.001	2.51 ± 0.09	1.72 ± 0.03	201.0 ± 0.9	328.8 ± 0.9	18.6 ^a^ ± 1.5	412.7 ± 2.4
	DP	0.371 ^a^ ± 0.003	1.040 ± 0.019	2.50 ± 0.04	1.63 ± 0.02	209.7 ± 5.7	366.9 ± 8.5	15.3 ^b^ ± 0.2	441.0 ± 10.4

For each foodstuff within the columns and each parameter, values with different letters are significantly different (*p* < 0.05). Values without letters or with the same letter are not significantly different (*p* > 0.05); Average ± (SD) (n = 3); DA—dry-ashing; MW—microwave; DP—graphite block acid digestion.

**Table 6:** Classification of digestion methods based on the criteria developed to evaluate the comparability with microwave digestion method.

Mineral	Quinoa		Amaranthus		Buckwheat	
	DA	DP	DA	DP	DA	DP
Cu	C	A	C	A	R	C
Mn	A	C	A	A	A	A
Fe	A	C	A	A	A	A
Zn	A	A	C	A	A	A
Mg	A	A	A	A	A	A
P	A	A	A	A	A	A
Ca	A	A	A	A	A	C
K	A	A	A	C	A	A
Na	<LOD	<LOD	<LOD	<LOD	<LOD	<LOD

DA—dry-ashing; DP—graphite block acid digestion; A—method is acceptable; C—method should be used with caution; <LOD—below the limit of detention.

## Data Availability

The original contributions presented in the study are included in the article, further inquiries can be directed to the corresponding author.
